# Antimicrobial and immune modulatory effects of lactic acid and short chain fatty acids produced by vaginal microbiota associated with eubiosis and bacterial vaginosis

**DOI:** 10.3389/fphys.2015.00164

**Published:** 2015-06-02

**Authors:** Muriel Aldunate, Daniela Srbinovski, Anna C. Hearps, Catherine F. Latham, Paul A. Ramsland, Raffi Gugasyan, Richard A. Cone, Gilda Tachedjian

**Affiliations:** ^1^Centre for Biomedical Research, Burnet InstituteMelbourne, VIC, Australia; ^2^Department of Microbiology, Nursing and Health, Faculty of Medicine, Monash UniversityClayton, VIC, Australia; ^3^Department of Infectious Disease, Monash UniversityMelbourne, VIC, Australia; ^4^Department of Immunology, Monash UniversityMelbourne, VIC, Australia; ^5^Department of Surgery Austin Health, The University of MelbourneHeidelberg, VIC, Australia; ^6^School of Biomedical Sciences, CHIRI Biosciences, Curtin UniversityPerth, WA, Australia; ^7^Department of Biophysics, Johns Hopkins UniversityBaltimore, MD, USA; ^8^Department of Microbiology and Immunology, The University of Melbourne at the Peter Doherty Institute for Infection and ImmunityParkville, VIC, Australia

**Keywords:** lactic acid, short chain fatty acids, bacterial vaginosis, lactobacilli, vaginal microbiota, metabolites, microbiome

## Abstract

Lactic acid and short chain fatty acids (SCFAs) produced by vaginal microbiota have reported antimicrobial and immune modulatory activities indicating their potential as biomarkers of disease and/or disease susceptibility. In asymptomatic women of reproductive-age the vaginal microbiota is comprised of lactic acid-producing bacteria that are primarily responsible for the production of lactic acid present at ~110 mM and acidifying the vaginal milieu to pH ~3.5. In contrast, bacterial vaginosis (BV), a dysbiosis of the vaginal microbiota, is characterized by decreased lactic acid-producing microbiota and increased diverse anaerobic bacteria accompanied by an elevated pH>4.5. BV is also characterized by a dramatic loss of lactic acid and greater concentrations of mixed SCFAs including acetate, propionate, butyrate, and succinate. Notably women with lactic acid-producing microbiota have more favorable reproductive and sexual health outcomes compared to women with BV. Regarding the latter, BV is associated with increased susceptibility to sexually transmitted infections (STIs) including HIV. *In vitro* studies demonstrate that lactic acid produced by vaginal microbiota has microbicidal and virucidal activities that may protect against STIs and endogenous opportunistic bacteria as well as immune modulatory properties that require further characterization with regard to their effects on the vaginal mucosa. In contrast, BV-associated SCFAs have far less antimicrobial activity with the potential to contribute to a pro-inflammatory vaginal environment. Here we review the composition of lactic acid and SCFAs in respective states of eubiosis (non-BV) or dysbiosis (BV), their effects on susceptibility to bacterial/viral STIs and whether they have inherent microbicidal/virucidal and immune modulatory properties. We also explore their potential as biomarkers for the presence and/or increased susceptibility to STIs.

## Introduction

The lower female reproductive tract, specifically the vagina and ectocervix, is considered a formidable chemical and physical barrier to exogenous invading organisms, in part due to the structure of the stratified vaginal epithelium and the presence of cervicovaginal fluid (CVF). Eubiotic viscoelastic CVF acts as an effective lubricant, facilitates the trapping of exogenous organisms and, acts as an acidified medium in which there is an arsenal of antimicrobial molecules (antibodies, defensins etc.). Importantly, this mucosal layer (mucus and layers of dead epithelial cells) also enables the adhesion of mutualistic vaginal microbiota (Boris et al., 1998). In asymptomatic women, microbiota acidify the vagina through lactic acid-production whilst bacterial vaginosis-associated bacteria (BVAB) produce many short chain fatty acids (SCFAs) that contribute the development of a dysbiotic vaginal environment (Aroutcheva et al., 2001a,b; Valore et al., 2002; Yeoman et al., 2013). The study of asymptomatic reproductive-age women has revealed that vaginal microbiota are unique to each woman but are largely dominated by lactic acid-producing bacteria, most often from the *Lactobacillus* genus (Verhelst et al., 2004; Fredricks et al., 2005; Ravel et al., 2011). Despite the differences in bacterial composition of vaginal communities, it seems that acidifying the vaginal milieu via generation of organic acid metabolites, predominantly lactic acid, is a conserved function (Ravel et al., 2011; Gajer et al., 2012). Humans are unique among primates in supporting lactic acid-producing vaginal microbiota (Mirmonsef et al., 2012a; Yildirim et al., 2014), particularly lactobacilli, which are largely responsible for acidifying the vaginal milieu with ~110 mM of lactic acid by anaerobic glycolysis of glycogen released from shed and shedding vaginal epithelial cells (Stanek et al., 1992; Boskey et al., 1999, 2001). This ultimately acidifies the vagina to an average pH ~3.5 (Fox et al., 1973; O'Hanlon et al., 2013), with both D and L optical isomers of lactic acid that differ in their arrangement of the same chemical components around a central carbon. The D/L ratio of lactic acid isomers matches the ratio produced by lactobacilli cultured from the same vaginal sample of CVF (Boskey et al., 2001*)* strongly indicating that lactic acid produced by lactobacilli is responsible for vaginal acidity. In women with lactobacillus-dominated flora, the vaginal pH tightly correlates with lactic acid concentration in CVF (O'Hanlon et al., 2013) suggesting that lactic acid is likely responsible for generating the protective acidic environment considered to be so important for antimicrobial activity.

Bacterial vaginosis (BV) is largely defined as a dysbiosis of the vaginal microbiome and is the most common and enigmatic vaginal condition in reproductive-age women. BV is characterized by a loss of lactic acid-producing bacteria and an increase in the number and diversity of anaerobic bacteria. Symptoms include an elevated vaginal pH >4.5 and malodorous abnormal discharge (Spiegel et al., 1980; Ling et al., 2010). BV is commonly diagnosed using two methods, the Amsel criteria or the Nugent Gram stain scoring method. A positive diagnosis of BV requires that three of the four Amsel criteria be satisfied; thin homogenous discharge, pH >4.5, amine odor and shed epithelial cells covered with bacteria (clue cells), whereas the Nugent Gram stain scoring method enumerates bacterial morphotypes associated with health (Gram-positive lactobacilli) and BV-associated morphotypes (Gram-variable rods). A low Nugent score (0–3) is indicative of healthy microbiota and a high Nugent score (7–10) is a positive diagnosis for BV (Nugent et al., 1991). Using this method, women diagnosed with BV may have a high Nugent score in the presence (symptomatic BV) or the absence (asymptomatic BV) of the aforementioned symptoms. BV risk factors commonly include menses, sexual activities and hygiene practices (Schwebke et al., 1999; Jespers et al., 2012; Srinivasan et al., 2012; Yeoman et al., 2013). BV is estimated to affect 5–15% Caucasian women, 20–30% Asian women, ~30% of Hispanic women, and 45–55% of Black women (Sewankambo et al., 1997; Schneider et al., 1998; Allsworth and Peipert, 2007; Bhalla et al., 2007; Fang et al., 2007; Koumans et al., 2007; Oliveira et al., 2007; Akinbiyi et al., 2008; Thoma et al., 2011). This is mirrored by global BV prevalence estimates showing a general trend toward high prevalence in Africa and low prevalence in Europe and Asia (Kenyon et al., 2013). BV plays a significant role in public health due to is association with various reproductive sequelae (Hillier et al., 1995; Donders et al., 2000; Ness et al., 2005; Leitich and Kiss, 2007) and increased susceptibility to sexually transmitted infections (STIs) including the human immunodeficiency virus (HIV) (Taha et al., 1998; Cu-Uvin et al., 2001; Cherpes et al., 2003; Wiesenfeld et al., 2003; Allsworth and Peipert, 2007; Coleman et al., 2007; Kaul et al., 2007; Atashili et al., 2008; Cohen et al., 2012).

Studies investigating the metabolites produced by lactobacillus-dominated microbiota and the polymicrobial BV states have observed a striking loss of lactic acid and a shift toward mixed SCFA (SCFA) production during BV (Al-Mushrif et al., 2000; Mirmonsef et al., 2011, 2012c; Gajer et al., 2012; O'Hanlon et al., 2013; Yeoman et al., 2013). Generally, acetate is the predominant metabolite in the CVF of women with symptomatic BV in addition to increased concentrations of propionate, butyrate and succinate (Stanek et al., 1992; Chaudry et al., 2004). Lactic acid and SCFA metabolites found in CVF have reported microbicidal, virucidal, and immune modulatory activities indicating their potential as biomarkers of disease and/or disease susceptibility (Al-Mushrif et al., 2000; O'Hanlon et al., 2011; Mirmonsef et al., 2012c; Aldunate et al., 2013).

## The vaginal microbiota

Albert Döderlein first described the presence of Gram-positive bacilli and low vaginal pH in the CVF of healthy reproductive-age women in 1892 (Döderlein, 1892). Later known as the *Lactobacillus acidophilus* complex (Thomas, 1928), lactobacilli came to be regarded as the foundation of healthy vaginal microbiota. In contrast, a paucity of lactobacilli and an increase in other bacterial morphotypes could be associated with symptomatic vaginal discharge (Döderlein, 1892; Curtis, 1914; Spiegel et al., 1980; Amsel et al., 1983; Jokipii et al., 1986). Many of the concepts of what could be considered healthy or unhealthy were based on distinctions made from microscopy and culture-dependent techniques; however, many also acknowledged that fastidious microbiota members were unlikely to be cultured. Nonetheless, the paradigm that lactobacilli equate to a healthy vaginal ecosystem became established and the true complexity of the microbiome would remain obscure until the advent of culture-independent molecular techniques.

### Cross-sectional studies of the vaginal microbiome in healthy asymptomatic women

Early cross-sectional studies revealed that the microbiota of asymptomatic reproductive-age women are indeed dominated by lactic acid-producing bacteria, primarily *Lactobacillus* (Verhelst et al., 2004; Zhou et al., 2004, 2007; Fredricks et al., 2005; Oakley et al., 2008). New members of the vaginal microbiome were also identified including *Atopobium* and *Lactobacillus iners* (Burton and Reid, 2002; Zhou et al., 2004). Later, Ravel et al. (2011) employed next generation sequencing in their landmark study to characterize the vaginal microbiomes of 396 ethnically diverse women of reproductive-age, revealing five core microbiomes termed community state types (CSTs). Four CSTs were described according to the dominant *Lactobacillus* species; *L. crispatus, L. gasseri, L. iners*, and *L. jensenii* corresponding to CST I, II, III, and V (Table 1). Collectively, *Lactobacillus* was the dominant species in 73% of the vaginal communities in this study group of healthy women. These four *Lactobacillus* species appear to represent the most prevalent bacteria in reproductive-age women, consistent with many previous investigations (Table 1) (Zhou et al., 2009, 2010; Forney et al., 2010; Ravel et al., 2011; Yoshimura et al., 2011; Martin et al., 2012; Smith et al., 2012). Importantly, this study also highlighted that 27% of women had communities characterized by microbial diversity assigned to CST IV (Ravel et al., 2011). Here, two subgroups IV-A or IV-B contained modest or no lactobacilli, respectively, in addition to several anaerobic bacteria known to produce lactic acid (Table 1). These community members are primarily strict or facultative anaerobes some of which are traditionally associated with dysbiosis, as in BV and include; *Atopobium, Gardnerella Prevotella, Mobiluncus, Megasphaera, Dialister, Sneathia, Streptococcus, Pseudomonas, Leptotrichia*, and *Aerococcus* amongst others. Although there is some debate as to whether these women are actually healthy and not asymptomatic for BV, it is worth noting a multitude of previous studies using various molecular methods also identified at least one group in their phylogenetic analyses that is analogous to the microbially diverse CST IV (Kim et al., 2009; Yamamoto et al., 2009; Forney et al., 2010; Zhou et al., 2010; Jespers et al., 2012). Therefore, it seems that bacteria traditionally associated with BV due to a high bacterial load, are likely members of eubiotic vaginal communities that have been overlooked in the past (Antonio et al., 1999; Pavlova et al., 2002; Zhou et al., 2004; Biagi et al., 2009; Yamamoto et al., 2009). Recent studies now show that 20–30% of women have diverse microbiomes not dominated by *Lactobacillus* that are not classically considered normal or healthy (Zhou et al., 2004, 2007, 2010; Srinivasan and Fredricks, 2008; Schellenberg et al., 2009; Ravel et al., 2011, 2013; Gajer et al., 2012).

**Table 1:** **Microbial communities and metabolite spectrum in the vaginal ecosystem and their proposed relationship to risks of adverse sexual and reproductive outcomes**.

**Community State Type (CSTs)**		**pH**	**Metabolite profile**
I	*L. crispatus*	4.0^a^	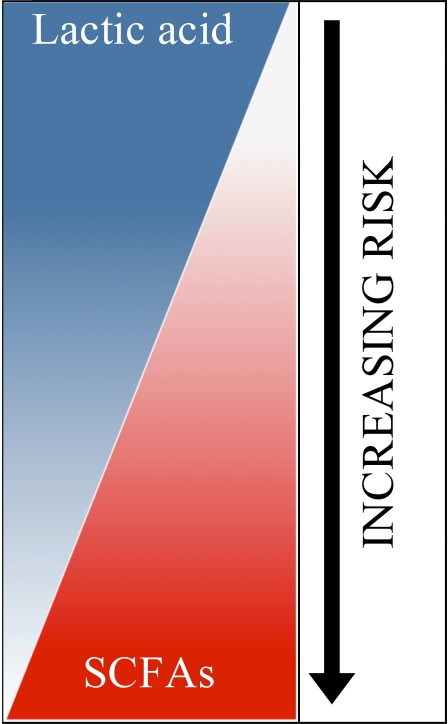
II	*L. gasseri*	5.0^a^	
V	*L. jensensii*	4.7^a^	
III	*L. iners* (ubiquitous regardless of BV status)	4.4^a^	
IV-A	Modest levels of *L. crispatus, L. iners* or other *Lactobacillus sp*. with low proportions of *Streptococcus, Anaerococcus, Corynebacterium* and *Finegoldia*	5.3^a^	
IV-B	Relatively high levels of *Atopobium, Gardnerella, Mobiluncus, Peptoniphilus, Sneathia, Prevotella* and several other taxa of BVAB		
BV	Polymicrobial Increased diversity and bacterial load of BVAB	>4.5	

a *pH was measured using a VpH glove test strip with pH determined according to color chart (Ravel et al., 2011) pH is likely to be lower if measured under vaginal hypoxic conditions with a pH electrode (O'Hanlon et al., 2013)*.

### Temporal studies of the vaginal microbiome and community dynamics using molecular characterization

Numerous cross-sectional studies have helped revise our understanding of bacterial communities in the vaginal ecosystem. However, this ecosystem is not static, there are many disturbances through continual efflux of CVF containing bacteria, antimicrobials, and sloughed epithelial cells from constant renewal of the vaginal epithelium. There are also cyclical changes in estrogen, glycogen content, pH and menses as well as the introduction of exogenous bacteria due to human activities such as sex and hygiene practices (Srinivasan et al., 2010; Gajer et al., 2012; Yeoman et al., 2013). Therefore, studies that analyze samples from a single time point merely offer a glimpse of the microbiome as it is affected by and responds to these intrinsic and extrinsic disturbances. The dynamic nature of the vaginal ecosystem can be assessed with temporal longitudinal studies that complement cross-sectional findings by evaluating shifts in community composition and structure, and their stability over time.

Overall, longitudinal studies reveal that the temporal dynamics of vaginal microbiota is unique to each reproductive-age woman (Santiago et al., 2011; Gajer et al., 2012; Hickey et al., 2013), with some possessing relatively stable communities (Wertz et al., 2008; Biagi et al., 2009; Santiago et al., 2011; Gajer et al., 2012; Jespers et al., 2012) and others experiencing brief albeit dramatic shifts (Brotman et al., 2010b; Gajer et al., 2012; Ravel et al., 2013). Altered community dynamics were commonly associated with menses (Srinivasan et al., 2010; Santiago et al., 2011; Gajer et al., 2012; Jespers et al., 2012; Hickey et al., 2013), sex (Srinivasan et al., 2010; Jespers et al., 2012; Santiago et al., 2012), and bacterial composition within a CST (Verstraelen et al., 2009; Gajer et al., 2012). Conversely, a high level of estrogen alone or estrogen and progesterone were associated with greater community stability (Gajer et al., 2012); this effect was also seen in pregnant women with high estrogen levels (Romero et al., 2014).

Recent studies have highlighted that shifts in vaginal community composition seem to be preferential (Verstraelen et al., 2009; Gajer et al., 2012; Smith et al., 2012; Romero et al., 2014). *L. crispatus* (CST I, Table 1) has been reported to experience few transitions to other CSTs and seems to be quite stable and promote vaginal community stability (Verstraelen et al., 2009; Gajer et al., 2012). *L. crispatus* is common in Caucasian and Asian women and less so in Black and Hispanic women (Zhou et al., 2004; Ravel et al., 2011; Jespers et al., 2012), and is present primarily in healthy asymptomatic women whilst *L. iners* is ubiquitous, even in women with dysbiosis such as BV (Verhelst et al., 2004; Zozaya-Hinchliffe et al., 2008; Biagi et al., 2009). It is therefore not surprising that microbiomes dominated by *L. iners* (CST III, Table 1) exhibit the greatest variation in composition and stability. One study reported that *L. iners* was twice as likely to transition to the diverse CST IV-B than IV-A (Gajer et al., 2012) meanwhile another concluded that *L. gasseri/L. iners* signify a predisposition to the occurrence of dysbiotic vaginal bacteria (Verstraelen et al., 2009). However, little is known about *L. gasseri* and *L. jensenii* and non lactobacillus-dominated microbiomes (CST IV, Table 1), because they are less prevalent in reproductive-age women. The observed shifts in vaginal communities has led to the proposition of an ecological hypothesis describing the maintenance of equilibrium in the vagina by transitioning between alternative communities whereby not all are favorable or conducive to maintaining community function (Ravel et al., 2011; Gajer et al., 2012).

### Role of lactic acid producing vaginal microbiota in the maintenance of community function

Previous studies have cited the production of lactic acid as a major conserved characteristic maintained through the functional redundancy exhibited by members of a transitioning community (Zhou et al., 2004; Linhares et al., 2010; Ravel et al., 2011; Gajer et al., 2012). This finding lends credence to the notion that several lactic acid-producing microbiota act to conserve community function and that variability in community composition is not necessarily a precursor to dysbiosis that leads to pathology. The vagina is subject to many disturbances; it is therefore not surprising that the ecosystem has evolved to accommodate changes in community composition through divergent organisms that perform a common function since vaginal homeostasis is more likely to be maintained. The range of lactic acid-producing bacteria commonly found in the vagina may therefore represent a group of particularly adept allelopathic organisms that use acid fermentation products, especially lactic acid, to suppress or otherwise outcompete other organisms and interact with the host in a manner that favors their competition. It follows that different community types may have varying levels of functionally redundant microbiota that maintain community performance; hence may exhibit varying degrees of resilience in the face of disturbances that could influence susceptibility to disease when combined with host determinants (Table 1). The incredibly complex and dynamic nature of the microbiome brings to light the numerous obstacles that must be surmounted by researchers who seek to uncover factors that elicit changes in the microbiome and consequently maintain eubiosis or lead to a dysbiotic state associated with risks of disease. It also highlights the need to move away from merely identifying what organisms are present toward determining their metabolic capacity, antimicrobial, immune modulatory effects, and function in the microbiome.

### Immune modulation by the vaginal microbiome

The vaginal mucosal epithelium acts both as a physical barrier and immunological mediator providing the first defense against potential infections (Kaushic, 2011; Anderson et al., 2014). Mucosal epithelia generally comprise multiple layers of rarely keratinized stratified squamous epithelium that rests on a lamina propria where the uppermost apical layers lack tight junctions (Blaskewicz et al., 2011; Anderson et al., 2014). These layers are permeable to water, soluble proteins, viruses, and penetrable by vaginal microbiota as well as cellular (e.g., CD4^+^ T cells and macrophages) and molecular mediators of the immune system (e.g., cytokines) (Blaskewicz et al., 2011; Carias et al., 2013; Politch et al., 2014). The vagina and endocervix provide immunological defenses by conferring tolerance to microbes, maintaining epithelial integrity, and recruiting and supporting immune cells (Fichorova and Anderson, 1999). As the front line of immune defense, epithelial cells express pattern recognition receptors (PRRs) including Toll-like receptors (TLRs) that respond to microbe- or pathogen-associated molecular patterns (MAMPs/PAMPs) (Table 2) by secreting cytokines and chemokines, antimicrobial peptides and other immune factors (Fichorova and Anderson, 1999; Herbst-Kralovetz et al., 2008; Kaushic, 2011; Rose et al., 2012). The pro-inflammatory response elicited by pathogens is normally required to control infections (Sandler et al., 2014). However, vaginal mucosal inflammation can promote transmission of viral STIs, such as HIV, by compromising epithelium integrity (Nazli et al., 2010) and by recruiting and activating HIV target cells (Li et al., 2009). Epithelial cell-derived immune mediators have pivotal roles in cell recruitment, immune regulation and tissue repair (Fichorova and Anderson, 1999). Given the intimate contact between the microbiota and their acid metabolites with the vaginal epithelium it is important to study how these interactions modulate mucosal immunity.

**Table 2:** **The effects of lactic acid and BV-associated SCFAs on TLR responses**.

**PRR**	**Common MAMPs/PAMPs**	**Organism recognized**	**Effect of lactic acid or SCFAs**
TLR1^a^,^b^			ND
TLR2^a^,^b^	Lipopeptides, lipopolysaccharide, peptidoglycan, flagellin	Bacteria	Acetate and butyrate induced production of pro-inflammatory cytokines IL-8, TNFα and IL-1β from human PBMCs and potentiated pro-inflammatory responses to TLR2 ligands^c^
TLR6^a^,^b^			ND
TLR3^a^,^b^	Double stranded ribonucleic acid (ds RNA)	Virus	In the VK2/E6E7 human vaginal epithelial cell line, L-lactic acid and poly I:C treatment led to the production of the pro-inflammatory immune mediators IL-8 and IL-1β in a standard tissue culture plate setup^d^.
TLR4^a^,^b^	Lipopolysaccharide Lipoteichoic acid Viral envelope proteins Protozoal phospholipids	Gram negative bacteria Gram positive bacteria Virus *Trichomonas vaginalis*	L-lactic acid and lipopolysaccharide increased IL-23 production from human PBMCs, may lead to preferential stimulation of Th17 T-lymphocytes that protect mucosa from extracellular bacteria^e^
TLR5^a^,^b^	Flagellin	Bacteria	ND
TLR7^a^,^b^	Single stranded ribonucleic acid (ss RNA)	Virus	Acetate and butyrate induced the production of pro-inflammatory cytokines of IL-8, TNFα and IL-1β from human PBMCs and potentiated pro-inflammatory responses to TLR7 ligands^c^
TLR8^a^,^b^			ND
TLR9^a^,^b^	Un-methylated components of nucleic acid	Bacteria Virus	ND

*ND-Not determined*.

a *Nasu and Narahara (2010)*.

b *Herbst-Kralovetz et al. (2008)*.

c *Mirmonsef et al. (2012c)*.

d *Mossop et al. (2011)*.

e *Witkin et al. (2011)*.

**Figure 1:**
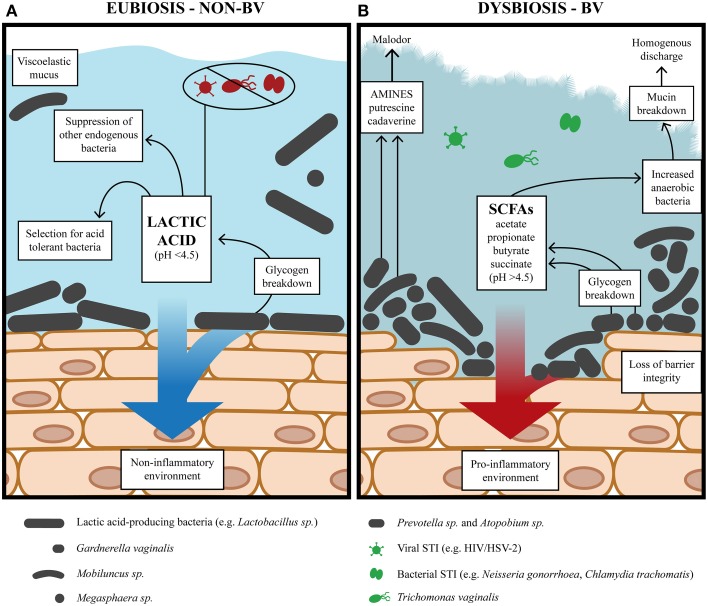
**The vaginal environment during alternative states of eubiosis and BV. (A)** During eubiosis, lactic acid-producing bacteria acidify the vaginal milieu pH <4.5 (average ~3.5) with lactic acid as the predominant metabolite. Lactic acid potently inactivates STIs while lactic acid-producers, such as *Lactobacillus*, generate a non-inflammatory environment. **(B)** During BV, the vaginal environment has a lower redox potential conducive to the growth of diverse anaerobic bacteria and higher bacterial load. The concentration of mixed SCFAs and amines also increase and is accompanied by loss of vaginal acidity pH >4.5. The diverse anaerobic bacteria generate virulence factors which compromise epithelial barrier integrity, degrade mucin and generate a pro-inflammatory environment.

Lactic acid-producing bacteria are known to maintain homeostasis by attenuating inflammation in the gut and by preserving gut barrier function (Zeuthen et al., 2008; Donato et al., 2010; Santos Rocha et al., 2012; Castillo et al., 2013; Perez-Santiago et al., 2013). Vaginal microbiota are reported to mediate immune modulatory effects on the vaginal mucosa (Al-Mushrif et al., 2000; Sakai et al., 2004; Nikolaitchouk et al., 2008; Joo et al., 2011; Kyongo et al., 2012). Studies in women from distinct geographical regions colonized with lactobacillus-dominated microbiota suggest an absence of vaginal inflammation, which may in part depend on the dominant *Lactobacillus* species. Specifically, vaginal lactobacilli are associated with lower levels of pro-inflammatory cytokines in CVF (e.g., IL-1α and/or IL-8) (Sakai et al., 2004; Nikolaitchouk et al., 2008; Kyongo et al., 2012), and in the case of *L. iners*, higher levels of secretory leukocyte peptidase inhibitor (SLPI) (Nikolaitchouk et al., 2008), an antimicrobial peptide normally depleted in women with dysbiosis such as BV (Draper et al., 2000; Mitchell et al., 2009; Balkus et al., 2010; Dezzutti et al., 2011). The presence of *L. crispatus* and *L. jensenii* were negatively associated with cellular inflammatory markers (Kyongo et al., 2012). In contrast, a study in adolescents failed to show differences for most cytokines in CVF from young women with dysbiosis compared to women with lactobacillus-dominated microbiota (Alvarez-Olmos et al., 2004), although further studies in distinct cohorts are required. Largely consistent with *in vivo* observations, *in vitro* studies demonstrate that vaginal lactobacilli are generally non-inflammatory when cultured on vaginal epithelium. *L. jensenii*, and *L. crispatus* do not elicit a pro-inflammatory response from vaginal epithelial cells (Figure 1A) (Libby et al., 2008; Fichorova et al., 2011; Eade et al., 2012; Rose et al., 2012; Yamamoto et al., 2013; Doerflinger et al., 2014). *L. iners and L. jensenii* induce PRR-signaling but this does not lead to an increase in secretion of pro-inflammatory mediators IL-6 and IL-8 (Yamamoto et al., 2013; Doerflinger et al., 2014). Although lactobacilli contain MAMPs that could potentially activate TLRs, *L. crispatus* and *L. jensenii* significantly dampen cytokine expression of IL-6, IL-8, and TNFα from vaginal epithelial cells following addition of exogenous TLR agonists (Rose et al., 2012). This suggests a role for commensal bacteria in maintaining vaginal homeostasis through as yet unknown mechanism(s) for modulating cytokine responses. Since these studies have largely focused on individual bacterial species, further investigations are required to fully elucidate the mucosal immune responses associated with the more complex CSTs dominated by distinct lactobacilli including the specific effects of lactic acid, as well as longitudinal studies to account for the dynamic nature of the vaginal microbiota (Gajer et al., 2012).

## Bacterial vaginosis, an ecological disorder

BV is a dysbiosis of the vaginal microbiota in reproductive-age women with unknown etiology and poorly understood pathogenesis. It is not known whether the bacteria commonly associated with the syndrome are a cause or a consequence of dysbiosis and unclear whether abnormal colonization is due to an exogenous pathogen, opportunistic endogenous microbiota, or if dysbiosis is triggered by vaginal microbiota unable to maintain community function in the face of particular disturbances. For women with lactobacillus-dominated microbiota, there is a depletion of lactobacilli and an overgrowth of anaerobic BV-associated bacteria (BVAB) (Figure 1B). The roles of different lactobacilli in preventing or promoting the transition to dysbiosis are an increasing area of investigation. As previously mentioned, an *L. crispatus*-dominated microbiota rarely transitions to CST IV-B (Table 1) containing BVAB, is not commonly found in women with BV and is associated with a more acidic vaginal milieu (Ravel et al., 2011; Gajer et al., 2012; O'Hanlon et al., 2013). Conversely, an *L. iners*-dominated microbiota is encountered in women regardless of BV status and may offer less protection from progression to BV. In this respect, a recent study by Macklaim et al. (2013) found that *L. iners* differentially expressed 10% of its genome when the vaginal ecosystem transitioned to dysbiosis. Further investigation is required to determine if *L. iners* is not antagonistic toward BVAB or less so than other lactobacilli, why *L. iners* seems tolerant of dysbiosis and if it contributes to dysbiosis.

### Bv-associated bacteria (BVAB)

As well as a paucity of lactobacilli, BV is accompanied by increased diversity and bacterial load of Gardnerella, Atopobium, Prevotella, Megasphaera, Dialister, Sneathia, Leptotrichia, Mobiluncus, Streptococcus, Bacteroides, Mycoplasma, Clostridiales BVAB 1, 2, 3, and, several other bacteria (Fredricks et al., 2005; Wertz et al., 2008; Kim et al., 2009; Verstraelen et al., 2009; Ling et al., 2010; Zozaya-Hinchliffe et al., 2010; Jespers et al., 2012; Santiago et al., 2012; Srinivasan et al., 2012). BVAB increase vaginal pH by generating mixed SCFAs and amines, and are also capable of utilizing lactic acid as an energy source (Figure 1B) (Spiegel et al., 1980; Wolrath et al., 2001; Macklaim et al., 2013; Yeoman et al., 2013). Many of these BVAB are common inhabitants of a eubiotic vaginal ecosystem but little is known of synergistic or antagonistic effects between them, with only Gardnerella and Prevotella displaying a synergism noted by several studies (Biagi et al., 2009; Ling et al., 2010; Zozaya-Hinchliffe et al., 2010; Ravel et al., 2011; Jespers et al., 2012; Datcu et al., 2013). Furthermore, different isolates may represent different strains/sub-strains that might be associated with BV. For example, different isolates of Atopobium have been shown to be resistant to metronidazole, the antibiotic used to treat BV (Ferris et al., 2004a,b; De Backer et al., 2006). BV isolates of *G. vaginalis* also have significant differences in genome content and gene order that have variable metabolic and virulence capacity (Harwich et al., 2010; Yeoman et al., 2010; Macklaim et al., 2013). Interestingly, dispersed and cohesive forms of *G. vaginalis* have also been identified, with the latter cohesive form associated with the initiation and formation of biofilms hypothesized to contribute to BV pathogenesis (Patterson et al., 2010; Swidsinski et al., 2010). Atopobium and *L. iners* have also been implicated in the formation of these biofilms (Swidsinski et al., 2005, 2008, 2010; Machado et al., 2013). However, further investigations are required to elucidate the contribution of various organisms in BV.

### Altered immune modulation during BV

While BV does not cause overt clinical inflammation characterized by redness and pain, it is nevertheless characterized by a pro-inflammatory response in the vaginal mucosa (Schwebke and Weiss, 2002; Mitchell and Marrazzo, 2014), which is likely to have distinct characteristics based on the bacterial communities responsible for BV. The cervicovaginal immune response in women with BV has been examined in several studies, which are reviewed in Mitchell and Marrazzo (2014). Overall pro- and anti-inflammatory cytokine levels tend to be elevated in women with BV (Cherpes et al., 2008). However, the levels of specific cytokines and antimicrobial peptides vary among studies, which have been proposed to be due to several factors including differences in study population, the bacterial communities responsible for BV, and the cross-sectional design and small sample sizes. Nevertheless, in most studies the pro-inflammatory cytokine, IL-1β is elevated and the SLPI antimicrobial peptide is decreased in women with BV while elevation of IL-6 and IL-8 is inconsistent among studies (Mitchell and Marrazzo, 2014). In addition, vaginal bacteria can modulate the innate immune response elicited by epithelial cells with species-specific immune signatures (Libby et al., 2008; Rose et al., 2012; Doerflinger et al., 2014). The BVAB *G. vaginalis* and *A. vaginae*, when cultured on vaginal or cervical epithelial cell monolayers and tissue models, elicit a pro-inflammatory response by up-regulating the production of cytokines (IL-6, IL-1β, TNFα, IL-8) and chemokines (e.g., RANTES, MIP-1β), some antimicrobial peptides (i.e., hBD-2, defensins) and membrane-associated mucins (Figure 1B) (Libby et al., 2008; Eade et al., 2012; Rose et al., 2012; Doerflinger et al., 2014). These responses are similar to what is observed in women with BV (Schwebke and Weiss, 2002; Hedges et al., 2006; Valore et al., 2006; Mitchell and Marrazzo, 2014). In contrast, *Prevotella bivia*, another BVAB, failed to elicit a pro-inflammatory response when cultured on vaginal epithelium (Doerflinger et al., 2014) indicating distinct pathogenicity of BV bacteria.

### The need to improve diagnosis of BV

The Amsel criteria and the Nugent score are the two main methods for BV diagnosis. The Amsel criteria, contains four criteria, at least three of which must be satisfied for a diagnosis of BV; a vaginal pH >4.5, thin grayish homogenous discharge, an amine odor upon addition of 10% potassium hydroxide to vaginal discharge and the presence of sloughed epithelial cells coated with BVAB (clue cells) in a Gram stained wet mount (Amsel et al., 1983). In contrast, the Nugent score is based on a Gram stain scoring method and is determined by assessing *Lactobacillus* morphotypes present as large Gram-positive bacilli, Gram-variable bacilli indicative of *G. vaginalis* morphotypes and curved Gram-variable bacilli indicative of *Mobiluncus* morphotypes (Nugent et al., 1991). A Nugent score of 7–10 is a positive diagnosis of BV. The Nugent scoring method is reported to have superior reproducibility and sensitivity relative to the Amsel criteria (Sha et al., 2005a; Modak et al., 2011); however, current diagnosis methods are biased in that they rely on the simplistic concept that lactobacilli equate to healthy vaginal microbiota and assessment occurs at a single time point. Our current understanding of the microbiome reveals that 20–30% of reproductive-age women have diverse lactic acid-producing microbiota that are not lactobacilli and that vaginal microbiota can experience dramatic transitions in community composition over time without transitioning to dysbiosis (Zhou et al., 2004, 2007, 2010; Ravel et al., 2011; Gajer et al., 2012). Consequently, current diagnostics may have confounded our current understanding of BV. Nonetheless, these are the best currently available diagnostic methods and in the future they are likely to be replaced by molecular quantification and characterization as technology advances.

### BV treatment and recurrence

In most women, BV will spontaneously resolve without treatment (Hay et al., 1997; Koumans et al., 2007; Laghi et al., 2014) however, treatment is recommended for symptomatic women due to potential BV-associated complications. Current recommended regimens include oral metronidazole (500 mg) twice a day for 7 days, metronidazole gel (0.75%) once a day for 5 days or clindamycin cream (2%) for 7 days, in conjunction with abstinence from sex or the use of condoms (CDC, 2010). Antibiotic treatment is often efficacious but to varying degrees in different women (Srinivasan et al., 2010). Recurrence is frequently inevitable and because the etiology of BV is unknown, it is unclear whether recurrence is due to antibiotic resistance, re-inoculation, re-emergence of endogenous bacteria, or some other disturbance leading to the loss of microbiota community function. Estimates indicate that BV will recur in 30% of women within 3 months of antibiotic treatment and >50% of women within 6 months (Hanson et al., 2000; Sanchez et al., 2004; Sobel et al., 2006; Bradshaw et al., 2006a,b; Nyirjesy et al., 2007).

A number of studies have attempted to address the cause of recurrent BV and suggest that antibiotic resistance and biofilms may play a role (Beigi et al., 2004; Verstraelen et al., 2004; Austin et al., 2005; Swidsinski et al., 2005, 2008, 2010)as well as extravaginal reservoirs (El Aila et al., 2009; Marrazzo et al., 2012; Petricevic et al., 2012; Santiago et al., 2012). Also, antibiotic treatment of BV can severely disrupt the microbiota (Santiago et al., 2012) and does not ensure that the microbiota will return to its pre-BV state (Agnew and Hillier, 1995; Nyirjesy et al., 2007). This suggests that whilst treatment may reduce the abundance of BVAB the consequent ecological void may allow for the re-emergence of opportunistic endogenous bacteria or unimpeded re-inoculation with exogenous pathogens. The antibiotic rifaximin is reported to overcome the limitations of existing antibiotic therapy (Cruciani et al., 2012, 2013; Donders et al., 2013) by restoring the vaginal community as well as the metabolome (Laghi et al., 2014). Alternative strategies for BV treatment such as oral and topical probiotics, which also aim to restore vaginal community function, are undergoing evaluation. However, the data are currently inconclusive, likely due to heterogeneity in clinical trial methodology and study endpoints, the administration route (oral/intravaginal), dosage, and strains of probiotic bacteria used and if they were provided as a cocktail or a single strain as well as the characteristics of the study populations (Senok et al., 2009; Mastromarino et al., 2013; Huang et al., 2014).

It is also important to consider that due to the nature of BV diagnosis methods, such as the Nugent score, women with non lactobacillus-dominated microbiota (CST IV) may have been misdiagnosed with BV and this may contribute to apparent BV recurrence and explain spontaneous BV resolution. Furthermore, the high rates of BV recurrence are not surprising when considering that the composition and abundance of BVAB in each woman exhibits great variation (Fredricks et al., 2005; Ravel et al., 2013; Yeoman et al., 2013) yet treatments are not targeted to accommodate these differences. With current technological advances it should be possible to stratify women according to the profile of BVAB and tailor therapy for better treatment outcomes.

### BV-associated complications and sequelae

BV is of clinical significance due to the multitude of adverse reproductive outcomes and increased risk of acquiring STIs. BV is associated with preterm birth (Eschenbach et al., 1984; Gravett et al., 1986; Kurki et al., 1992; Riduan et al., 1993; Hay et al., 1994; Holst et al., 1994; McGregor et al., 1994; Hillier et al., 1995; Wennerholm et al., 1997), pelvic inflammatory disease (Ness et al., 2005), spontaneous abortion (Donders et al., 2000), post-abortal infection (Larsson et al., 2000), and miscarriage (Hay et al., 1994; Llahi-Camp et al., 1996; Ralph et al., 1999; Oakeshott et al., 2002; Nelson et al., 2007); indicating that BV may predispose women to abnormal ascending colonization of the upper reproductive tract. BV is also associated with an elevated risk of acquiring STIs including *Neisseria gonorrhea* (Morris et al., 2001; Wiesenfeld et al., 2003; Brotman et al., 2010a; Gallo et al., 2012), *Chlamydia trachomatis* (Morris et al., 2001; Wiesenfeld et al., 2003; Schwebke and Desmond, 2007; Brotman et al., 2010a; Gallo et al., 2012), *Trichomonas vaginalis* (Martin et al., 1999; Brotman et al., 2010a, 2012), herpes simplex virus type-2 (HSV-2) (Cherpes et al., 2003; Kaul et al., 2007; Nagot et al., 2007), human papilloma virus (HPV) (Watts et al., 2005; Gillet et al., 2011) as well as HIV (Sewankambo et al., 1997; Taha et al., 1998; Cu-Uvin et al., 2001; Coleman et al., 2007; Atashili et al., 2008; Cohen et al., 2012).

BV may facilitate the acquisition of STIs, through a variety of mechanisms stemming from the production of factors and metabolites that create a favorable environment for the growth other organisms (Hedges et al., 2006; Beigi et al., 2007; Rose et al., 2012; Yeoman et al., 2013; Doerflinger et al., 2014) whilst reducing the capacity to inactivate exogenous pathogens due to a loss of lactic acid and lactic acid-producing microbiota (Figure 1B). In this regard, BVAB have been shown to increase vaginal pH which would enable the survival of acid-labile exogenous pathogens including most of the aforementioned STIs a woman with BV is at increased risk of contracting (McGrory et al., 1994; Brotman et al., 2010a; Graver and Wade, 2011; Aldunate et al., 2013; Gong et al., 2014). In addition to increased acquisition risks, the altered dynamics of the vaginal ecosystem during BV (Figure 1B) may also facilitate the sexual transmission of STIs, with several studies noting increased viral replication and viral load in the CVF of women with HIV-1 and HSV-2 (Cu-Uvin et al., 2001; Cherpes et al., 2005; Cohn et al., 2005; Sha et al., 2005b; Coleman et al., 2007; Mitchell et al., 2013) and transmission of HIV to their male partners (Cohen et al., 2012). In contrast, women with vaginal microbiota dominated by lactic acid-producing bacteria negatively correlate with BV acquisition (Skarin and Sylwan, 1986; Tamrakar et al., 2007; Srinivasan and Fredricks, 2008; Zozaya-Hinchliffe et al., 2010) and have a reduced risk of acquiring STIs including *N. gonorrhea* (Martin et al., 1999; Wiesenfeld et al., 2003), *C. trachomatis* (Wiesenfeld et al., 2003), *T. vaginalis* (Martin et al., 1999; Brotman et al., 2012), and HIV (Martin et al., 1999).

Overall, several studies denote that acidic vaginal conditions and lactic acid-producing microbiota are associated with reduced susceptibility to STIs whereas BV, with an elevated pH and reduction in lactic acid-producing bacteria, appears to increase susceptibility (Figure 1). However, the maintenance of the vaginal ecosystem and the development of dysbiosis remain a mystery and the degree of protection afforded by distinct vaginal communities requires further investigation. Furthermore, BV symptoms are varied amongst women and these may reflect different etiologies and or different pathogenesis mechanisms leading to BV in the context of a woman's unique genetic determinants and microbiota. The characterization of microbiota members has certainly enhanced our understanding of the vaginal ecosystem but supplementary information regarding microbiota metabolic activities and their impact on the function and performance of the vaginal ecosystem is required to gain a complete understanding of vaginal eubiosis and dysbiosis.

## The metabolome in eubiosis and dysbiosis

During the reproductive years the vaginal microbiome provides a multifaceted primary defense mechanism against infections whilst promoting favorable reproductive outcomes. One manner in which this is achieved is the production of acid fermentation products, primarily lactic acid, which acidifies the vagina through the concerted effort of lactic acid-producing bacteria. Thus, the production of lactic acid, together with host factors, deeply impacts the resulting microbiota by excluding or selecting community members (Figure 1A). Collectively, these members generate the functional output of that particular community thereby influencing resilience in the face of intrinsic and extrinsic disturbances. A representation of both host and microbial factors in the vaginal ecosystem is captured within CVF. The metabolome, in particular SCFAs and amines, are the most extensively studied components of this complex medium.

### Early studies of CVF metabolites associated with eubiosis and BV

Early studies investigated SCFAs and amines in CVF through a variety of chromatographic techniques in an effort to identify metabolites associated with dysbiosis that could be used for the diagnosis of a polymicrobial syndrome, then known as non-specific vaginitis (NSV) (Gardner and Dukes, 1955; Spiegel et al., 1980; Piot et al., 1982; Ison et al., 1983). The criteria later established by Spiegel (Spiegel et al., 1983), Amsel (Amsel et al., 1983), and Nugent (Nugent et al., 1991), determined that the vast majority of NSV cases were actually BV. Overall, early studies in healthy asymptomatic reproductive-age women found that lactic acid was the predominant metabolite in CVF with relatively small amounts of the SCFAs acetate, propionate, butyrate, isobutyrate, succinate, formate, fumarate, valerate, and caproate (Preti and Huggins, 1975; Spiegel et al., 1980; Piot et al., 1982; Ison et al., 1983; Krohn et al., 1989; Stanek et al., 1992) as well as trace amounts, if any, of the amines putrescine, cadaverine, and spermidine (Chen et al., 1979, 1982; Sanderson et al., 1983; Jones et al., 1994; Kubota et al., 1995; Wolrath et al., 2001). In contrast, BV-associated metabolite profiles showed that lactic acid was often dramatically decreased along with an increased concentration and variety of SCFAs and amines that were more prevalent in women with BV (Spiegel et al., 1980; Piot et al., 1982; Ison et al., 1983; Jokipii et al., 1986; Krohn et al., 1989; Stanek et al., 1992; Chaudry et al., 2004) as reviewed in Wolrath et al. (2001).

The BV-associated SCFA profiles obtained using gas liquid chromatography (GLC) prompted the development of a BV marker based on the correlation of a high succinic:lactic acid ratio and dysbiosis determined by fulfilling two of the following criteria; pH >4.5, homogenous discharge, presence of clue cells and amine odor (Spiegel et al., 1980). A peak ratio of = 0.4 showed 86% sensitivity, 97% specificity for BV and 90% positive predictive value, where the sensitivity could be increased to 90% if propionate and butyrate peaks were detected. This method was used with varying success to diagnose BV, with most studies reporting 80–90% sensitivity and 80–97% specificity (Piot et al., 1982; Ison et al., 1983; Gravett et al., 1986; Krohn et al., 1989). Early studies also sought to assess changes in metabolite profiles associated with the antibiotic treatment of BV and consistently found a return to increased lactic acid with a concomitant decrease in BV-associated SCFAs acetate, propionate, butyrate, isobutyrate, succinate and caproate and, other organic acids (Spiegel et al., 1980; Stanek et al., 1992; Chaudry et al., 2004) as well as BV-associated amines (Chen et al., 1979, 1982; Sanderson et al., 1983). Thus, the relative levels of lactic acid and SCFAs in CVF often reflected the success or failure of antibiotic treatment.

A paucity of lactobacilli in conjunction with increased bacterial diversity and load was associated with altered metabolite profiles of women with BV vs. those of healthy asymptomatic women (Piot et al., 1982; Jokipii et al., 1986). Many BVAB are known to produce several acid metabolites; for example: *Bacteroides* (succinate-producers), *Peptococcus* (butyrate- and acetate-producers), *Clostridium* and Gram-positive cocci (caproate-producers) and, *Dialister* (propionate-producer) (Spiegel et al., 1980; Debrueres and Sedallian, 1985; Downes et al., 2003; Chaudry et al., 2004). Particular attention was paid to *G. vaginalis* (acetate and succinate-producer), because of the work of Gardner and Dukes (1955). However, *G. vaginalis* was frequently cultured from women regardless of BV status and the detection and quantity of acetate- or succinate was not associated with *G. vaginalis* (Spiegel et al., 1980; Piot et al., 1982). Holmes et al. (1985) employed an alternative approach to ascertain whether the altered metabolic profile was due to an unknown host determinant or microbial metabolism. Here, the redox potential (E*_h_*) at the vaginal epithelium was measured in healthy asymptomatic women and women with BV, it was hypothesized that vaginal microbiota would influence the redox potential due to altered concentrations of organics acids that form redox pairs (e.g., lactate/pyruvate and succinate/fumarate) (Holmes et al., 1985). Women with BV were reported to have a more reduced vaginal environment compared to healthy asymptomatic women. Typically, redox potential varies by ± 60 mV per pH unit but the difference between the two groups was 262 mV for 1 pH unit difference, indicating that pH alone could not account for the altered redox potential. The difference in redox potential was attributable to microbial metabolism rather than a host determinant because antibiotic treatment successfully increased the redox potential, similar to that of healthy asymptomatic women. Notably, a more reduced vaginal environment is conducive to the growth of anaerobes, which increase in number and diversity during BV. However, as with other BV-associated changes, this study could not conclude whether the reduced environment was a cause or consequence of dysbiosis.

It was hoped that important insights would be obtained into microbial and host interactions during BV by assessing CVF in eubiosis and dysbiosis. However, no clear association could be made between specific BVAB and ensuing changes in metabolite profiles. Nonetheless, these studies contributed to revealing BV as a multifaceted syndrome associated with gross metabolic changes in addition to dysbiotic microbiota and altered immune regulation.

### Recent studies of CVF metabolites associated with eubiosis and BV

Advances in molecular technology and metabolite detection are now supplementing these early studies by characterizing the complexity of a vaginal community, shared metabolic pathways of community members and the metabolic output of microbiota. As mentioned previously, the temporal dynamics of the vaginal microbiota demonstrates that shifts in community members is not necessarily a precursor to dysbiosis because some community members exhibit functional redundancy and are able to maintain lactic acid production (Gajer et al., 2012). For example, Gajer et al. (2012) reported two women (Subjects 22 and 28) that maintained lactic acid production despite a transition from *L. gasseri* to *Streptococcus* or *L. crispatus* to *L. iners*. Conversely, long-term changes in vaginal microbiota were associated with a depletion of lactobacilli and an increase in BVAB with decreased lactic acid and increased levels of succinate and acetate (Subjects 8 and 10). In this regard, ensuing molecular characterization has shed some light on the relative importance of different lactobacilli as well as taking into consideration the contribution of other lactic acid-producing bacteria.

A study by Bai et al. (2012) employing pyrosequencing of the 16S rRNA gene to determine microbial composition and proton nuclear magnetic resonance (^1^H NMR) spectroscopy for the metabolome, confirmed that lactic acid was the predominant metabolite in women with microbiomes dominated by lactobacilli. Generally, this study showed that 89% of women with diverse microbiota resembling CST IV (Table 1) had metabolic end-products in CVF consisting of 3–12% lactic acid vs. women with *L. crispatus*-dominant microbiomes with 18–27% lactic acid. However, it is thought that lactobacilli differ in their metabolic output and relative contribution of lactic acid since the abundance of lactic acid did not appear to correlate with the abundance of a particular *Lactobacillus* species. This study showed that *L. crispatus* dominated microbiomes had some of the highest levels of lactic acid and is consistent with previous studies where *L. crispatus* has been associated with a more acidic vaginal milieu (Ravel et al., 2011; O'Hanlon et al., 2013). However, different levels of SCFAs were also observed in microbiomes with different community composition, belonging to the same CST. For example subject SO6 and SO4 both harbored CST I; SO6 was dominated almost exclusively by *L. crispatus* with high lactic acid levels while SO4 was dominated by *L. crispatus, L. iners*, and *L. jensenii* and was accompanied by higher levels of acetate and succinate. *L. gasseri*-dominated microbiomes were associated with lower levels of lactic acid, consistent with the findings of Ravel et al. (2011) citing a higher pH in CST II (Table 1). Interestingly, subject SO2 harbored a microbiome with high Nugent score composed of *L. iners, Prevotella, Megasphaera*, and *Lachnospiraceae* and high levels of acetate and succinate; yet, the levels of lactic acid produced were marginally higher than that of subject SO1 with a low Nugent score and a microbiome dominated by *L. gasseri*. These findings highlight the variable metabolic output of different lactobacilli species as well as demonstrating functional redundancy regarding lactic acid production.

Alternatively, Yeoman et al. (2013) employed metabolomics and a network-based approach to link particular metabolites with vaginal community members in women with BV. Here, the loss of lactic acid was the single most distinguishing feature of women diagnosed with BV using the Nugent method. Interestingly, BV was not identified as a single entity but rather two subtypes; symptomatic BV type I (SVBI) associated with high levels of acetate and high Nugent scores, and SBVII associated with high levels of propionate and intermediate or low Nugent scores. Major determinants for SBVI and SBVII were identified as derivatives of lactic acid including 1,2-propanediol and acetate both resulting from the degradation of lactic acid and 1-acetoxy-2-propanol, which can be obtained via lipase activity from 1,2-propanediol (Izquierdo et al., 2000; Oude Elferink et al., 2001). Overall, a network-based approach implicated several BVAB in BV symptomology, including malodor with the presence of amines that were linked to *Dialister*, 2-methyl-2-hydroxybutyric acid associated with discharge and linked to *Mobiluncus* and diethylene glycol associated with pain which was associated with *G. vaginalis*. Collectively, this study concurs with previous suggestions that the etiologies and pathogenesis mechanisms of BV may vary depending on the bacteria and metabolites present in the context of a woman's unique vaginal environment.

It is important to note that metabolic analyses are limited by the ability to detect metabolites that are present in appreciable amounts and primarily constitute the end-products of metabolic pathways. Therefore, the important roles and interaction of intermediate metabolites and pathways are not fully appreciated and will likely require the combination of metabolomics with metagenomics in future investigations. One such approach is meta-RNA-seq employed by Macklaim et al. (2013) with the aim of analyzing the differential expression of genes and pathways in vaginal microbiota during eubiosis and dysbiosis. This study assessed two healthy asymptomatic women, N4 and N30, with different vaginal community types both largely dominated by *L. crispatus* and two women, B27 and B31, with diverse vaginal communities diagnosed with BV according to the Nugent score. Subject B27 harbored microbiota dominated by *L. iners* followed by *L. crispatus, Prevotella*, and *Gardnerella* while B31 contained *Prevotella, L. iners, Gardnerella*, and *Megasphaera*. When the gene expression of healthy asymptomatic women vs. women with BV was analyzed by gene function in relation to linked metabolic pathways, the two states were found to largely differ in pathways for energy metabolism. In healthy asymptomatic subjects dominated by *L. crispatus*, there was an up-regulation of steps in the glycolytic pathway responsible for lactic acid-production and lactic acid was predicted as the predominant metabolite. In contrast, changes associated with BV included the up-regulation of steps in the tri-carboxylic acid cycle and oxidative phosphorylation as well as the up-regulation of the enzymes butryl-coA-dehydrogenase and butyrate kinase, which are predicted to increase succinate and butyrate, respectively. Furthermore, many BVAB increased the expression of enzymes presumably involved in carbohydrate/glycogen uptake and metabolism. In addition, enzymes expressed by *Prevotella amnii, Dialister*, and *Megasphaera* were linked to amine production. Overall, in healthy asymptomatic women who harbored *L. crispatus* as the dominant organism, microbiota metabolism was largely oriented toward the production of lactic acid that is responsible for generating a protective acidic environment (Figure 1A) (Macklaim et al., 2013; O'Hanlon et al., 2013). In contrast, women with BV harbored less lactobacilli and diverse microbiota geared toward the production of succinate and butyrate as well as amines. Together these would generate a vaginal environment with low redox potential conducive to the growth of anaerobes as well as increased vaginal pH, potentially allowing the survival of exogenous acid-labile pathogens and or allow the growth of opportunistic endogenous microbiota (Figure 1B).

Recent studies and cumulative knowledge over several decades consistently show a dramatic loss of lactic acid in BV, as well as increased prevalence and production of acetate, propionate, butyrate and succinate, and amines (Table 3). Further investigation is required to elucidate how these metabolites are involved in microbiota and host interactions and their roles in community function. This knowledge will be essential for understanding dysbiosis and associated risks of disease (Table 1) as well as determining appropriate prevention and therapy for certain BVAB and metabolic profiles. In this regard, the application of metagenomics holds great promise particularly for a multifaceted polymicrobial syndrome such as BV, which has resisted conventional analysis.

**Table 3:** **Shifts in CVF metabolite concentration in eubiosis compared to the CVF of women with BV**.

**Acid/SCFA concentration during eubiosis [mM]**		**Acid/SCFA concentration during BV [mM]**	
Lactic	~120*^a^,^b^,^e^*	Lactate	<20*^e^*
Acetic	0–4*^b^,^c^,^d^,^f^*	Acetate	<120*^a^,^c^,^d^,^f^*
Propionic	<1	Propionate	2–4*^c^*
Butyric	<1	Butyrate	2–4*^c^*
Succinic	<1*^e^*	Succinate	<20*^e^*

a *Gajer et al. (2012)*.

b *O'Hanlon et al. (2013)*.

c *Mirmonsef et al. (2011)*.

d *Mirmonsef et al. (2012c)*.

e *Al-Mushrif et al. (2000)*.

f *Chaudry et al. (2004)*.

## The antimicrobial properties of lactic acid and SCFAs produced by vaginal microbiota in eubiosis and dysbiosis

Lactic acid is the predominant acid metabolite found in the CVF of healthy asymptomatic women Table 3 (Preti and Huggins, 1975; Spiegel et al., 1980; Piot et al., 1982; Ison et al., 1983; Krohn et al., 1989; Stanek et al., 1992; Bai et al., 2012; Macklaim et al., 2013; Yeoman et al., 2013). Glycogen, and its breakdown products (glucose and maltose) produced by the action of vaginal α-amylase (Spear et al., 2014), is thought to be the primary energy source used by lactobacilli during anaerobic glycolysis that leads to lactic acid production (Figure 1A) (Paavonen, 1983; Wilson et al., 2010). Further, high free glycogen levels and lactobacilli in the vaginal lumen tend to correlate with low vaginal pH (Mirmonsef et al., 2014). In stark contrast, glycogen and lactic acid are depleted in women with BV (Mirmonsef et al., 2012a), possibly due to increased glycogen consumption by BVAB that would prevent regrowth of lactic acid-producing bacteria (Figure 1B) (Mirmonsef and Spear, 2014).

### Lactic acid is a potent microbicide under hypoxic vaginal conditions but hydrogen peroxide (H_2_O_2_) is not

Acidification of the vagina by lactic acid was originally proposed as the primary antimicrobial mechanism by which lactobacilli defend against reproductive tract pathogens (Döderlein, 1892). However, the production of H_2_O_2_ by H_2_O_2_-generating lactobacilli was subsequently favored as a prominent antimicrobial factor present in the CVF of healthy asymptomatic women (Klebanoff and Coombs, 1991; Klebanoff et al., 1991). This was based on studies reporting that the prevalence of H_2_O_2_-producing lactobacilli is lower in women with BV (Eschenbach et al., 1989) and that H_2_O_2_-producing lactobacilli inactivate HIV (Klebanoff and Coombs, 1991) and BVAB (Klebanoff et al., 1991) While H_2_O_2_ is present at low concentrations in CVF, its ability to inhibit microbes in the context of the reducing and hypoxic vaginal environment has recently been cast into serious doubt (O'Hanlon et al., 2010, 2011; Graver and Wade, 2011; Gong et al., 2014). Specifically, studies with BVAB, *N. gonorrhea* and *C. trachomatis* demonstrate that the primary microbicidal activity under hypoxic vaginal conditions is due to lactic acid and not H_2_O_2_ (O'Hanlon et al., 2010; Graver and Wade, 2011; Gong et al., 2014).

**Figure 2:**
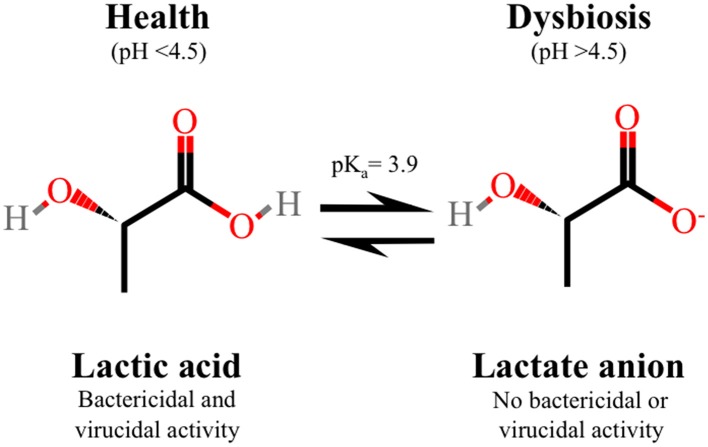
**Protonated lactic acid exists in equilibrium with the un-protonated lactate anion, which is determined by the acid dissociation constant (pK_a_ 3.9)**. In healthy asymptomatic women of reproductive age with low vaginal pH, lactic acid predominates which is the microbicidal and virucidal form with immune modulatory activity. In contrast, the lactate anion, which does not have microbicidal or virucidal activity, predominates in women with bacterial vaginosis with a neutral vaginal pH.

It was only recently that the key role of lactic acid in vaginal acidification was convincingly demonstrated (O'Hanlon et al. (2013). Here, 64 CVF samples obtained from 56 women with lactobacillus-dominated flora as determined by low Nugent scores (0–3), were analyzed to reveal a strong inverse correlation between vaginal pH and lactic acid concentration. Quantitation of physiological concentrations of lactic acid and pH, measured under conditions recapitulating the hypoxic vaginal environment, demonstrated an average pH of 3.5 ± 0.3 (mean ± SD) with a range of 2.8–4.2, and an average total lactic acid concentration of 1.0% ± 0.2% w/v (O'Hanlon et al., 2013). These data differ markedly from previous studies that reported a higher eubiotic vaginal pH of 4.2 (Owen and Katz, 1999). Overall, this study demonstrates that lactic acid, in conditions that resemble a eubiotic vaginal environment, has a major role in acidifying the vagina to pH values lower than previously described. Lactic acid can exist as protonated lactic acid or the unprotonated lactate anion, which is determined by the acid dissociation constant (pK_a_ 3.9) (Figure 2). Under neutral conditions, the lactate anion predominates whereas in an acidic vaginal milieu, undissociated lactic acid predominates and it is this form that is endowed with microbicidal and virucidal activity (O'Hanlon et al., 2011; Aldunate et al., 2013). Given that lactic acid is more abundant at lower pH and taken together with the latest physiological levels of lactic acid and vaginal pH (O'Hanlon et al., 2013), the microbicidal form of lactic acid is calculated to be 11-fold more concentrated in women with lactobacillus-dominated microbiota and likely more important as a microbicide than otherwise indicated by prior research.

### Lactic acid potently inactivates BV-associated bacteria

Many pathogens are well recognized as being susceptible to acid but the potential role of lactic acid in the vaginal ecosystem and its specific antimicrobial effects have not been extensively characterized. The *in vitro* studies performed by O'Hanlon et al. (2011) demonstrate that lactic acid at physiological concentrations (55–111 mM) and at pH 4.5 inactivates 17 different species of BVAB including *G. vaginalis* and *Atopobium vaginae* while not affecting the viability of the four main vaginal lactobacilli, *L. jensenii, L. gasseri, L. crispatus*, and *L. iners*, even at a concentration 1110 mM (10% w/v) that is 10 times above physiological levels (O'Hanlon et al., 2011). A potent ~10^6^-fold decrease in viability was observed for all BVAB treated with 111 mM (1% w/v) lactic acid, pH 4.5 for 2 h at 37°C (O'Hanlon et al., 2011). In contrast, treatment with lactic acid at pH 7.0 is not microbicidal indicating that the activity is pH-dependent and is mediated by protonated lactic acid not the lactate anion (Figure 2) (O'Hanlon et al., 2011). Lactic acid is significantly more potent in inactivating BVAB compared to media acidified to pH 4.5 with an inorganic acid (i.e., HCl) or with acetic acid at concentrations corresponding to physiological levels of lactic acid (55–111 mM) (O'Hanlon et al., 2011).

Both lactic- and acetic acids are weak membrane permeant acids (Kashket, 1987). However, acetic acid is smaller and more protonated than lactic acid at an equivalent pH, due to its higher acid dissociation constant (pK_a_ ~4.8). Therefore, it would be expected to acidify the bacterial cytosol and lead to cell death more rapidly. However, the antibacterial activity of lactic acid is markedly more potent than acetic acid suggesting that it might act by additional mechanisms such as disturbing bacterial membranes (Alakomi et al., 2000). The relatively weak antimicrobial activity of acetic acid is of importance due to its predominance over lactic acid during BV (Table 3) and suggests that the loss of lactic acid leads to altered competitive dynamics in the vaginal environment resulting in less inhibition of endogenous opportunistic bacteria and BVAB (Figure 1B).

### Lactic acid potently inactivates HIV

It is well recognized that HIV is acid-labile as determined using a variety of acids including HCl, phosphate/citrate buffers, acetic acid or lactic acid to inactivate virus (Martin et al., 1985; Ongradi et al., 1990; Kempf et al., 1991; O'Connor et al., 1995; Connor, 2006). However, only recently has the HIV virucidal activity of lactic acid at physiological concentrations been directly compared to other acids including acetic acid that predominates during BV. We have shown that at a typical vaginal pH of 3.8, 1% (w/w) L-lactic acid is significantly more potent and rapid in inactivating HIV_Ba−L_, a CCR5-coreceptor using laboratory strain, and transmitted-founder strains HIV-1_RHPA.c/2635_ and HIV-1_CH058.c/2960_, known to have established a productive infection in a new host (Keele et al., 2008; Salazar-Gonzalez et al., 2009), compared to 1% (w/w) acetic acid and acidity alone (HCl), all adjusted to pH 3.8 (Aldunate et al., 2013). In addition, lactic acid has broad-spectrum anti-HIV activity where complete inactivation of different HIV-1 subtypes and HIV-2 was achieved with ≥0.4% (w/w) L-lactic acid (Aldunate et al., 2013). L-lactic acid at 1% (w/w) also maximally inactivates HIV-1 in the presence of cervicovaginal secretions and seminal plasma (Aldunate et al., 2013). Like the BVAB lactic acid inactivation studies (O'Hanlon et al., 2011), the anti-HIV activity of L-lactic acid is abolished at neutral pH, indicating that lactic acid and not the lactate anion is virucidal (Figure 2). HIV-1 inactivation is irreversible since loss of infectivity is observed even after adjusting the pH of the medium to neutral following virus acid treatment (Aldunate et al., 2013).

Intriguingly, physiological levels of lactic acid at pH 4.0 do not disrupt viral particles (Lai et al., 2009). The superior potency of lactic acid compared to other acids is similar to findings with BVAB suggesting a common antimicrobial mechanism (O'Hanlon et al., 2011) i.e., permeabilization of the lipid membrane (Alakomi et al., 2000). However, the mechanism by which lactic acid inactivates HIV-1 is likely to be multifactorial and could include targeting of viral or virion-incorporated host proteins leading to loss of function by denaturation (Tang et al., 2003). In this regard, the HIV-1 virucidal activity of the L-lactic acid isomer is 17-fold greater than D-lactic acid at the threshold concentration of 0.3% (w/w) and pH 4.0 (Aldunate et al., 2013), suggesting a protein target. Apart from the anti-HIV activity of lactic acid, the presence of distinct ratios of the L- and D-lactic acid isomers in cervicovaginal mucus has been implicated as a surrogate marker for a vaginal microbiota factor that hinders and traps viral particles, which could impair the ability of the virus to reach target cells in the vaginal epithelium (Lai et al., 2009; Shukair et al., 2013).

Whilst knowledge about the antimicrobial activity of lactic- and acetic acid is increasing, very little is still known with regards to the antimicrobial activity of propioniate, succinate and butyrate, which are increased in concentration and prevalence in women with BV. So far, our findings suggest that a high level of vaginal lactic acid, at concentrations generated by eubiotic microbiota, is a potent virucide whereas SCFAs at BV-associated concentrations and pH do not lead to any detectable HIV inactivation (Aldunate et al., 2014). Demonstration of potent virucidal activity of lactic acid supports its role in defending against viral pathogens such as female-to-male and mother-to-child transmission of HIV (Frank et al., 2012) by reducing vaginal shedding of infectious virus. The virucidal activities of lactic acid may explain in part the inverse correlation observed between HIV genital shedding in women and the presence of *Lactobacillus* (Sha et al., 2005b; Borgdorff et al., 2014) although this needs to be proven. In contrast, the role of lactic acid in mitigating male-to-female transmission of HIV is not immediately obvious. The antiviral activity of lactic acid is pH-dependent and semen deposited into the vaginal lumen increases pH to neutral (Fox et al., 1973), which could attenuate the anti-HIV activity of lactic acid (Aldunate et al., 2013). Antiviral efficacy of lactic acid in this context would depend on the ability of resident lactobacilli to rapidly acidify the vaginal lumen (Boskey et al., 1999) before cell-free or cell-associated HIV are able to migrate through the mucous layer and squamous vaginal epithelium and ectocervix; or transcytose/transmigrate through the columnar epithelium of the endocervix to be able to reach target cells (Cone, 2014).

### Lactic acid potently inactivates HSV-2

Women with BV are also at increased risk of acquiring HSV-2 (Cherpes et al., 2003), which is a cofactor for HIV acquisition, suggesting that lactobacilli and their product(s) might have a role in blocking this viral STI. In this regard lactobacilli inhibit HSV-2 replication through multiple effects. These include inhibiting viral entry and replication, which is related to the adhesion capacity of the lactobacillus strain, and the secretion of lactic acid that has HSV-2 virucidal activity and also inhibits virus replication by a virucidal-independent mechanism (Dimitonova et al., 2007; Conti et al., 2009). Similar to HIV, physiological concentrations of lactic acid (55–111 mM) at low pH (4.0) but not neutral pH, cause potent and irreversible HSV-1 and HSV-2 inactivation (Conti et al., 2009; Isaacs and Xu, 2013). However, unlike HIV, culture medium acidified to the same pH with HCl produces similar levels of HSV-2 inactivation as lactic acid (Conti et al., 2009). Although this observation needs to be confirmed, it suggests that the HIV virucidal mechanism of lactic acid is distinct from HSV-2.

## Immune modulatory effects of lactic acid and SCFAs associated with eubiosis and dysbiosis

The immune modulatory effects of vaginal microbiota, that could prevent or promote susceptibility to infection by reproductive tract pathogens, may in part be mediated through the production of organic acid metabolites including lactic acid as well as acetate, propionate, butyrate, and succinate (Kantor et al., 2009; Ravel et al., 2011; Bai et al., 2012; Gajer et al., 2012; Das et al., 2015). The critical role of microbiota metabolites in shaping the host mucosal immune response and for limiting inflammation in the context of an environment densely colonized with bacteria has gained prominence in studies focused on the gastrointestinal mucosa (Maslowski et al., 2009; Arpaia et al., 2013; Furusawa et al., 2013; Arpaia and Rudensky, 2014). Similar to the gut, the vaginal mucosa is colonized with mutualistic bacteria. The metabolite composition of CVF may be indicative of microbiota with diverse taxonomic compositions but representing similar functional states that could be informative as biomarkers for distinct immune effects on the host. However, little is known about the immune modulatory effects of SCFA metabolites produced by vaginal microbiota in the context of the unique and acidic vaginal environment.

### Immune modulatory effects of lactic acid

Immune modulating properties have been ascribed to L-lactic acid (Shime et al., 2008; Mossop et al., 2011; Witkin et al., 2011). Studies in the cancer field demonstrate that L-lactic acid produced by tumor cells generates a mildly acidic microenvironment (pH 6.0–6.5) that promotes tumor survival and metastasis by directly mediating immunosuppressive effects via decreased cytotoxic T cell function, monocyte function, dendritic cell maturation, and cytokine production from immune cells (Gottfried et al., 2006; Fischer et al., 2007; Goetze et al., 2011; Choi et al., 2013). By contrast, in the presence of TLR2 and TLR4 ligands, L-lactic acid produced from tumors promotes the IL-23/IL-17 pro-inflammatory pathway in monocyte/macrophages (Shime et al., 2008). More recent studies reveal that treatment with L-lactic acid at 15 mM followed by lipopolysaccharide (LPS), a TLR4 agonist, increases the production of IL-23 from human PBMCs compared with LPS treatment alone and this may lead to preferential stimulation of Th17 T-lymphocytes (Table 2) (Witkin et al., 2011). Notably, induction of IL-23 was not observed when L-lactic acid was tested at neutral pH or when HCl was substituted for L-lactic acid indicating that potentiation of this cytokine was not due to acidity alone (Witkin et al., 2011). However, the pH values (7.0 to 6.4) these authors report for L-lactic acid concentrations ranging from 30 to 60 mM are inconsistent with our report that 30 mM lactic acid (~0.3% L-lactic acid) acidifies culture medium to pH 3.9 (Aldunate et al., 2013). All together, these studies suggest that in the context of conventional immune cells, L-lactic acid mediates anti-inflammatory or pro-inflammatory effects depending on the experimental conditions.

L-lactic acid also elicits immune mediator release from the human vaginal epithelial cell line, VK2/E6E7, in the presence of the viral TLR3 agonist polyinosinic acid: cytidylic acid [poly (I:C)] (Mossop et al., 2011) (Table 2). L-lactic acid (at 15 mM) and TLR3 ligand treatment increased the production of the pro-inflammatory immune mediators IL-8 and IL-1β in a standard tissue culture plate setup (Mossop et al., 2011). Once again, the effect was reported to be dependent on treatment with L-lactic acid at acidic pH, and was not recapitulated with acetic acid, another smaller acid metabolite that is produced by BVAB (Mossop et al., 2011). These effects appear to be at odds with the reported anti-inflammatory and non-inflammatory effects of vaginal lactobacilli (Kyongo et al., 2012; Rose et al., 2012; Yamamoto et al., 2013; Doerflinger et al., 2014). Therefore, we propagated the VK2/E6E7 epithelial cell line in transwell culture conditions (Hearps et al., 2014) and performed a direct comparison to standard tissue culture plate conditions described by Mossop et al. (2011). Our study demonstrates that 0.3% L-lactic acid at pH 3.9 causes total cell death when VK2/E6E7 cells were cultured in standard tissue culture plates (Hearps et al., 2014) suggesting that toxicity may contribute to altered cytokine production. Another plausible explanation for the disparate results in these two studies is that L-lactic acid confers distinct immune effects at levels approaching physiologic concentrations (55–111 mM) and pH found in a healthy asymptomatic vagina. Further work is needed to define the impact of lactic acid and SCFAs at physiologic pH found in eubiosis and dysbiosis on immune mediators produced by vaginal and cervical epithelial cells and how this affects susceptibility to reproductive tract infections.

### Immune modulatory effects of BV-associated SCFAs

The effect of SCFAs produced by gut microbiota on innate immune function and epithelial integrity has been extensively studied (Macia et al., 2012; Arpaia and Rudensky, 2014). In the gut, SCFAs have an anti-inflammatory role by suppressing the production of pro-inflammatory cytokines through inhibition of NF-κB signaling and histone deacetylase (HDAC) (Segain et al., 2000; Luhrs et al., 2002a,b; Tedelind et al., 2007; Cox et al., 2009; Maslowski et al., 2009; Arpaia and Rudensky, 2014). In stark contrast to the gut, SCFAs in the vagina appear to be pro-inflammatory in women with BV (Mirmonsef et al., 2012c). The SCFAs, acetate and butyrate, but not propionate at high concentrations (20 mM) tested at neutral pH induce the production of pro-inflammatory cytokines IL-8, TNFα, and IL-1β from human peripheral blood mononuclear cells (PBMCs) and potentiated pro-inflammatory cytokine responses to TLR2 and TLR7 ligands at low concentrations (≤2mM) (Table 2) (Mirmonsef et al., 2012c). The apparent discordant immune effects of SCFAs in the gut compared to BV-related studies could be ascribed to inherent differences in these organs including cell-type, SFCA concentration, and pH (Cavaglieri et al., 2003; Bailon et al., 2010; Mirmonsef et al., 2012c) which would alter the ratios of protonated vs. unprotonated forms of the acids that can have different biological functions (O'Hanlon et al., 2011; Witkin et al., 2011; Aldunate et al., 2013). In addition, the pro-inflammatory response elicited by BV-associated SCFAs is consistent with a similar pro-inflammatory response produced by BVAB when grown on vaginal epithelial cells *in vitro* (Libby et al., 2008; Eade et al., 2012; Rose et al., 2012; Doerflinger et al., 2014), indicating that these metabolites may contribute to the sub-clinical inflammatory state observed in women with BV.

While SCFAs appear to promote a pro-inflammatory response in PBMCs under defined physiological conditions that are relevant to BV, a very limited number of studies have attempted to explore their direct immune modulatory role in dysbiosis. Initial work has primarily assessed the effect of various SCFAs on neutrophil polymorphonuclear leukocytes (PMNLs), important for mounting an inflammatory response by recognition of chemical signals that enable their migration to the appropriate site for phagocytosis (Al-Mushrif et al., 2000). Notably, BV is characterized by sub-clinical inflammation accompanied with the absence of neutrophil PMNLs (Cauci et al., 2003). It was thought that a variety of SCFA metabolites could be virulence factors that inhibit neutrophil PMNL and monocyte migration thereby enabling the unrestricted growth and establishment of BVAB. Initial work assessed the BVAB *Bacteroides* a known succinate-producer and reported that succinate could inhibit both the chemotactic and phagocytic responses of neutrophil PMNLs (Rotstein et al., 1985). Likewise, another study assessed the effect of pure and BVAB derived lactate, acetate and succinate on a monocytic cell line (MonoMac 6) as well as human monocytes (Al-Mushrif et al., 2000). This study reported that succinate exhibited the highest inhibition of chemotaxis followed by acetate with significant but relatively less inhibition, in a dose dependent manner. In contrast lactic acid did not significantly inhibit monocyte chemotaxis. Similar trends were seen in both the monocytic cell line as well as fresh human monocytes. In addition, *Prevotella* and *Mobiluncus* broth cultures as well as CVF from women with BV both contained high concentrations of succinate and acetate that also demonstrated monocyte chemotaxis inhibition. In contrast, *Lactobacillus* broth cultures as well as the CVF from women with lactobacillus-dominated microbiota showed no inhibition of monocyte chemotaxis (Al-Mushrif et al., 2000). Overall, these studies suggest that SCFAs produced by BVAB may contribute to BV pathogenesis by acting as virulence factors that prevent the migration of immune cells capable of generating an inflammatory response and this may in turn allow the establishment of BVAB.

#### Immune modulatory effects of BV-associated SCFAs on HIV

Several studies have focused on the mechanisms by which the sub-clinical inflammation of BV and BV-associated SCFAs could increase the risk of acquiring STIs, with a particular focus on HIV. The main proposed mechanisms include altered innate mucosal immunity leading to reduced barrier integrity of the vaginal epithelium (Nazli et al., 2010) and the activation and recruitment of HIV target cells to the genital mucosa (Kantor et al., 2009; Thurman and Doncel, 2011; Mirmonsef et al., 2012b; Das et al., 2015). BVAB could promote a pro-inflammatory vaginal environment through the production of SCFAs (Mirmonsef et al., 2012c). In the case of HIV, SCFAs such as butyrate/butyric acid with known HDAC inhibitory activity (Imai et al., 2012), could promote establishment of infection in the vaginal mucosa through activation of transcriptionally silenced HIV proviral and episomal DNA (Kantor et al., 2009; Das et al., 2015) in resting CD4^+^ T cells, that mainly comprise the small founder population during the initial phases of HIV infection (Zhang et al., 1999; Haase, 2011). Succinate, another BV-associated metabolite, has been shown to increase HIV expression in monocytes infected with HIV_Ba−L_ and cells differentiated with either granulocyte-macrophage colony-stimulating factor (GM-CSF) or macrophage colony-stimulating factor (M-CSF) (Graham et al., 2013). The authors proposed that this might contribute to increased viral shedding in the CVF of HIV positive women with BV. In this study, succinate was also the only metabolite that increased IL-8 levels relative to lactic acid, propionate, and butyrate which may contribute to pro-inflammatory conditions (Graham et al., 2013). A pro-inflammatory milieu would also lead to activation and recruitment of HIV target cells to the founder population, amplification of virus replication in the lower reproductive tract, and subsequent virus spread to the periphery (Haase, 2011).

Since BV is associated with an increased risk of STI acquisition, eliminating BV could indirectly protect against STIs. This could be achieved by promoting vaginal colonization with lactic acid-producing bacteria that compete for binding sites on vaginal epithelium and that produce antimicrobial factors, including lactic acid, to selectively suppress the growth of BVAB (Joo et al., 2011; O'Hanlon et al., 2011). However, further investigation is warranted regarding the contribution of BV-associated SCFAs and their immune modulatory effect on the vaginal epithelium remains to be elucidated.

## Biomarkers for BV

Recently, our understanding of the complex vaginal ecosystem has evolved dramatically in regards to microbiota composition, metabolic output and related immunological changes in eubiosis and dysbiosis. Yet, by its current definition BV remains a relatively non-specific marker of risk for a multitude of adverse sexual and reproductive outcomes. To date, the limited studies that have sought to identify biomarkers specific for BV have primarily involved analysis of the CVF proteome in relation to outcomes of BV-associated reproductive sequelae (Balu et al., 2003; Dasari et al., 2007; Kolialexi et al., 2008; Shah et al., 2009). However, based on knowledge we have put forward in this review, we believe that bacterial metabolites distinct for alternative states of eubiosis and dysbiosis are excellent biomarker candidates and should be a focus of future studies regarding BV biomarker development.

Major differences in metabolite concentration are consistently associated with a eubiotic state largely dominated by lactic acid vs. dysbiosis characterized by decreased lactic acid and increased mixed SCFAs and amine production, as well as increased vaginal pH (Figure 1, Table 3) (Spiegel et al., 1980; Stanek et al., 1992; Wolrath et al., 2001; Ravel et al., 2011; Yeoman et al., 2013). Microbiota metabolites as biomarkers for BV have the advantage that they reflect changes in vaginal microbiota that are associated with BV but overcome the difficulty of attempting to link specific bacterial species/strains, with uncharacterized metabolic capacities that may be functionally redundant. Notably, attempts to correlate specific bacteria with the metabolic output of the polymicrobial microbiota in BV have not been successful in the past and will be complicated by the presence of diverse microbiota (CST IV) in reproductive-age women. Furthermore, the presence of increased mixed SCFAs can influence vaginal redox potential and may indicate a reduced vaginal environment that favors the overgrowth of BV-associated anaerobes in addition to signaling immunological changes in the vaginal ecosystem that may contribute to BV pathogenesis.

Currently, the different SCFA profiles associated with eubiosis and dysbiosis may have some utility as biomarkers that complement established BV diagnostic methods by identifying true cases of BV rather than women that are diagnosed with BV because they harbor a diverse non lactobacillus-dominated microbiota or because the microbiome was transitioning at the time when BV was diagnosed. In this respect, Gajer et al. (2012) showed that the transitioning of vaginal community composition was possible whilst maintaining lactic acid production and that only long-term changes in microbiota composition were associated with changes in metabolite profiles (Gajer et al., 2012). Furthermore, Laghi et al. (2014) assessed the metabolome of women diagnosed with BV and found that those with a high lactic acid content vs. BV-associated mixed SCFAs, at the time of a positive BV diagnosis, were likely to experience spontaneous remission in the absence of treatment, indicating that these were not true cases of BV. The application of metabolites as markers to determine the probability of spontaneous remission could identify women whose microbiota are in a state of transition but not necessarily transitioning to BV and would prevent the unnecessary use of antibiotics that may severely impact the microbiome and promote BV as well as contributing to antibiotic resistance. The assessment of metabolites in CVF may also be used to determine the efficacy of BV treatment and the probability that BV recurrence will occur by tracking whether lactic acid production has increased during and after antibiotic treatment and, could aid in determining whether a different antibiotic or regimen is required for better treatment outcomes. It has been shown that women that experienced BV persistence and BV recurrence did not have significantly increased lactic acid levels during or following treatment (Spiegel et al., 1980; Laghi et al., 2014).

It is envisioned that future studies will use vaginal metabolic profiles to identify distinct subtypes of BV and associated microbial groups/subsets. These biomarkers could be used for stratifying women with a particular type of metabolic profile of BV for treatment with targeted antibiotics as well as promoting the development of treatments tailored to each subtype. Indeed, one study by Yeoman et al. (2013) has already employed metabolomics to identify two distinct symptomatic BV metabotypes linked with certain organic acids and certain bacteria, implicated in BV symptomology. Meanwhile, metagenomics studies are shedding light on changes in bacterial metabolism in respective states of eubiosis and dysbiosis and are aiming to link these with the differential expression of genomic content in specific vaginal microbiota with common functional pathways (Macklaim et al., 2013). In addition, it is proposed that different metabolite profiles may not only be associated with but also contribute to increased or decreased periods of disease risk and reproductive complications over time (Table 1). The proper identification and treatment of these women would be a crucial step in improving their reproductive outcomes and reducing their risk of acquiring STIs.

## Conclusions

Presently, the development of metabolic biomarkers specific for BV requires further investigation and will be challenging given the heterogeneity of vaginal microbiota and research gaps regarding BV etiology and pathogenesis in addition to the role of host determinants in eubiosis and dysbiosis, which have not been extensively studied. Moreover, these metabolites will have to be distinct to each state of eubiosis and dysbiosis and be consistently present in women experiencing each state. Yet, to date there has been no large-scale study assessing BV-associated SCFAs in representative groups of reproductive-age women. Further investigation is also required to elucidate changes in metabolic pathways, redox homeostasis, and inflammatory pathways so that we may understand the biological implications of increased SCFA concentrations in the vaginal ecosystem. There are also likely to be a multitude of bacterial and host metabolites and antimicrobial factors in the vaginal milieu that contribute to protection or contribute to BV symptomology and pathogenesis, which will need to be elucidated.

The shift from a simplistic paradigm where lactobacilli equate to vaginal health, to a greater awareness of vaginal ecosystem complexity has in part been fuelled by advancing technologies enabling the study of polymicrobial syndromes, such as BV, that withstand conventional analysis. We now know that lactobacilli are the most prevalent species in reproductive-age women. However, there are a significant number of women that harbor diverse microbiota that may also maintain community performance. Cumulative interdisciplinary knowledge suggests that allelopathic lactic acid-producing microbiota are key species in shaping vaginal communities and maintaining community performance associated with decreased risk of adverse sexual and reproductive health outcomes. Importantly, the demonstration that lactic acid has inherent microbicidal, virucidal, and immune modulatory properties suggests that protection of the vaginal environment extends beyond a mere role of vaginal acidification. Lactic acid is present in high ~110 mM concentrations in eubiosis and is far more abundant than other antimicrobial peptides and protective factors that have been studied more extensively, and is a primary determinant of the state of the vaginal environment. Lactic acid certainly warrants further investigation particularly in relation to BV-associated SCFAs, which replace lactic acid, and appear to increase BV-associated risks. A more holistic understanding of BV is likely to spur new strategies that may block or modify certain BV-associated metabolites with a view to promote vaginal health and reinforce/maintain a protective vaginal ecosystem that will prevent the development of BV. Given the protective actions of lactic acid produced by vaginal microbiota, discovering how BVAB or host factors cause the loss of lactic acid-producing microbiota and episodes of BV, may be critical to developing methods that maintain eubiosis. Lastly, the potent virudical and microbicidal functions of lactic acid generated by lactic-acid producing vaginal microbiota, strongly suggest that strategies to promote and maintain lactic acid-producers such as lactobacilli, perhaps by sustained delivery of lactic acid or the effective administration of probiotic lactobacilli, may lead to improved sexual and reproductive health outcomes.

### Conflict of interest statement

Richard A. Cone has a pending grant application to develop an intravaginal ring for lactic acid. Gilda Tachedjian, Anna C. Hearps, Raffi Gugasyan, and Richard A. Cone have grant funding from National Health & Medical Research Council of Australia (1088564, 1028294) to study vaginal acids and Gilda Tachedjian and Anna C. Hearps are inventors on a provisional patent on lactic acid (P38667AUP1). The remaining authors declare the absence of any commercial or financial relationships that could be construed as a potential conflict of interest
